# Prognosis of ischemic stroke patients with both aortic atheroma and cardioembolic sources

**DOI:** 10.1038/s41598-024-60294-1

**Published:** 2024-06-03

**Authors:** Jae Wook Jung, Minyoul Baik, JaeWook Jeong, Il Hyung Lee, Kwang Hyun Kim, Jaeseob Yun, Chi Young Shim, Geu-Ru Hong, Young Dae Kim, Ji Hoe Heo, Hyo Suk Nam

**Affiliations:** 1 Department of Neurology, Yonsei University College of Medicine, 50-1 Yonsei-ro, Seodaemoon-gu, Seoul, 03722 Korea; 2 Department of Neurology, Yonsei University College of Medicine, Yongin Severance Hospital, Yongin, Korea; 3 Department of Cardiology, Yonsei University College of Medicine, Seoul, Korea

**Keywords:** Aortic atheroma, Transesophageal echocardiography, Cardioembolic stroke, Prognosis, Neurology, Neurological disorders, Stroke

## Abstract

This study aimed to investigate the relationship between complex aortic plaque (CAP) and short-term as well as long-term outcomes following cardioembolic stroke. CAP is a known risk factor for occurrence and recurrence of ischemic stroke. However, the association of CAP on cardioembolic stroke remains unclear. This was retrospective study using prospective cohort of consecutive patients with cardioembolic stroke who underwent transesophageal echocardiography. The functional outcome was evaluated using the modified Rankin Scale score at 3 months, and long-term outcomes were assessed by recurrence of ischemic stroke and occurrence of major adverse cardiovascular events (MACE). Among 759 patients with cardioembolic stroke, 91 (12.0%) had CAP. Early ischemic stroke recurrence within 3 months was associated with CAP (*p* = 0.025), whereas CAP was not associated with functional outcome at 3 months (odd ratio  1.01, 95% confidence interval [CI]  0.57–1.84, *p* = 0.973). During a median follow-up of 3.02 years, CAP was significantly associated with ischemic stroke recurrence (hazard ratio = 2.68, 95% CI 1.48–4.88, *p* = 0.001) and MACE occurrence (hazard ratio = 1.61, 95% CI 1.03–2.51, *p* = 0.039). In conclusion, CAP was associated with early ischemic stroke recurrence and poor long-term outcomes in patients with cardioembolic stroke. It might be helpful to consider transesophageal echocardiography for patients with cardioembolic stroke to identify CAP.

## Introduction

Aortic atheroma (AA) is a known risk factor for both occurrence and recurrence of ischemic stroke^1–3^. Complex aortic plaque (CAP), characterized by complicated morphologies or an aortic arch thickness of 4 mm or more, is strongly associated with recurrent stroke^3–5^. The poor prognosis of CAP may be associated with the lack of established treatments and may vary by etiology of stroke. While several studies have reported on the relationship between CAP and prognosis, research regarding the prognosis of patients with CAP according to stroke etiology is scarce^6,7^.

Our previous study showed that the presence of both cardioembolism and large artery atherosclerosis exhibited poorer long-term prognosis compared to those with isolated cardioembolism^8^. Furthermore, patients with multiple cardioembolic sources exhibited more severe clinical symptoms^9^. Similarly, CAP may serve as an additional risk factor that can influence the prognosis of cardioembolic stroke. Transesophageal echocardiography (TEE) is useful for detecting embolic sources in ischemic stroke. While patients with cardioembolism, particularly atrial fibrillation, are often diagnosed without TEE, omitting TEE assessment can leave CAP undetected.

In this study, we investigated the prognostic effect of CAP for short-term and long-term outcomes in cardioembolic stroke. We hypothesized that CAP would increase the risk of ischemic stroke recurrence and major adverse cardiovascular event (MACE) occurrence in patients with cardioembolic stroke.

## Results

### Study population and baseline characteristics

From January 2012 to December 2018, 4,465 patients with acute ischemic stroke and transient ischemic attack were consecutively enrolled. We excluded 167 patients with transient ischemic attack and 3,091 patients with ischemic stroke or diagnoses other than cardioembolism etiologies including 742 (16.6%) patients with large-artery atherosclerosis, 364 (8.2%) with lacunar infarction, 162 (3.6%) with stroke of other determined etiology, 804 (18.0%) with undetermined two or more causes identified, and 1,019 (22.8%) with stroke or undetermined negative evaluation. Additionally, we excluded 448 patients with cardioembolic stroke who did not undergo TEE. Finally, 759 patients were included in this study (Supplementary Fig. 1). Patients who underwent TEE were younger, more likely to be men, and less likely to have hypertension, diabetes mellitus, atrial fibrillation, previous stroke history, and endovascular treatment. Pre-stroke mRS 0–1 was more frequent in patients who underwent TEE (Supplementary Table 1).

Among the 759 patients with cardioembolic stroke, 438 (57.7%) had high-risk PCSE and 321 (42.3%) had medium-risk PCSE. There was no significant difference in the proportion of PCSE between study groups except for the variable of left atrial appendage thrombus. The most commonly detected high-risk PCSE was atrial fibrillation, while the most prevalent medium-risk PCSE was spontaneous echo contrast, with patent foramen ovale being the second most frequent. (Table 1). The median age was 68 years (IQR 57–75) and 310 (40.8%) were women. The median duration from admission to TEE was 4 days (IQR 3–6). CAP was found in 91 (12.0%) patients, who were older, more likely to be men, and more likely to have hypertension, diabetes mellitus, and a history of antiplatelet or statin therapies. There was no statistical difference in pre-stroke mRS score, initial NIHSS score, thrombolytic therapies, or endovascular thrombectomy between groups (Table 2).

**Table 1:** TOAST classification of high- and medium risk sources of cardioembolism according to the presence of CAP.

	Total (*n* = 759)	CAP ( +) (*n* = 91)	CAP (−) (*n* = 668)	*p-*value
High-risk PCSE				
Atrial fibrillation (except for lone atrial fibrillation)	402 (53.0)	55 (60.4)	347 (51.9)	0.128
Left atrium appendage thrombus	54 (7.1)	11 (12.1)	43 (6.4)	0.049
Akinetic LV segment	33 (4.3)	6 (6.6)	27 (4.0)	0.270
Mechanical prosthetic valve	28 (3.7)	2 (2.2)	26 (3.9)	0.563
Mitral stenosis with atrial fibrillation	16 (2.1)	2 (2.2)	14 (2.1)	0.999
Dilated cardiomyopathy	6 (0.8)	0 (0.0)	6 (0.9)	0.999
Myocardial infarction within 4 weeks	4 (0.5)	2 (2.2)	2 (0.3)	0.073
Sick sinus syndrome	4 (0.5)	0 (0.0)	4 (0.6)	0.999
Infective endocarditis	3 (0.4)	0 (0.0)	3 (0.4)	0.999
LV thrombus	1 (0.1)	0 (0.0)	1 (0.1)	0.999
Atrial myxoma	1 (0.1)	0 (0.0)	1 (0.1)	0.999
Medium-risk PCSE				
Spontaneous echo contrast	188 (24.8)	26 (28.6)	162 (24.3)	0.370
Patent foramen ovale	182 (24.0)	29 (31.9)	153 (22.9)	0.060
LV hypokinesia	84 (11.1)	10 (11.0)	74 (11.1)	0.980
Congestive heart failure	48 (6.3)	7 (7.7)	41 (6.1)	0.568
Atrial septal aneurysm	36 (4.7)	4 (4.4)	32 (4.8)	0.999
Mitral annular calcification	32 (4.2)	6 (6.6)	26 (3.9)	0.259
Atrial flutter	17 (2.2)	1 (1.1)	16 (2.4)	0.709
Mitral stenosis without atrial fibrillation	11 (1.4)	0 (0.0)	11 (1.6)	0.378
Bioprosthetic heart valve	7 (0.9)	0 (0.0)	7 (1.0)	0.999
Mitral valve prolapse	6 (0.8)	1 (1.1)	5 (0.7)	0.537
Nonbacterial endocarditis	4 (0.5)	0 (0.0)	4 (0.6)	0.999
High- and medium-risk sources of cardioembolism				0.055
Patients with high-risk PCSE	438 (57.7)	61 (67.0)	377 (56.4)	
Patients with medium-risk PCSE	321 (42.3)	30 (33.0)	291 (43.6)	

Values are presented as number (%).

CAP = complex aortic plaque; LV = left ventricle; PCSE = potential cardiac source of embolism; TOAST = Trial of Org 10,172 in Acute Stroke Treatment.

**Table 2:** Baseline characteristics of study population according to the presence of CAP.

	Total (*n* = 759)	CAP ( +) (*n* = 91)	CAP (−) (*n* = 668)	*p-*value
Demographic variables				
Age (years), median (IQR)	68 (57, 75)	75 (68, 79)	67 (55, 75)	< 0.001
Sex (women), n (%)	310 (40.8)	21 (23.1)	289 (43.3)	< 0.001
Hypertension	525 (69.2)	72 (79.1)	453 (67.8)	0.028
Diabetes	215 (28.3)	39 (42.9)	176 (26.3)	0.001
Dyslipidemia	119 (15.7)	18 (19.8)	101 (15.1)	0.251
Coronary artery disease	209 (27.5)	32 (35.2)	177 (26.5)	0.082
Malignancy	83 (10.9)	8 (8.8)	75 (11.2)	0.485
Atrial fibrillation	402 (53.0)	55 (60.4)	347 (51.9)	0.128
Current smoker	136 (17.9)	19 (20.9)	117 (17.5)	0.432
Cardiac rhythm monitoring	639 (90.5)	75 (89.3)	564 (90.7)	0.683
Previous ischemic stroke	104 (13.7)	16 (17.6)	88 (13.2)	0.251
Previous hemorrhagic stroke	27 (3.6)	4 (4.4)	23 (3.4)	0.553
Stroke characteristics				
Pre-stroke mRS (2–5), (%)	33.0 (4.3)	3.0 (3.3)	30.0 (4.5)	0.787
Initial NIHSS score, median (IQR)	3 (1, 7)	3 (1, 7)	3 (1, 8)	0.886
IV-tPA	94 (12.4)	9 (9.9)	85 (12.7)	0.441
Endovascular treatment	104 (13.7)	13 (14.3)	91 (13.6)	0.863
Antiplatelet, prior to admission	246 (32.4)	39 (42.9)	207 (31.0)	0.023
Anticoagulation, prior to admission	128 (16.9)	15 (16.5)	113 (16.9)	0.918
Statin, prior to admission	173 (22.8)	29 (31.9)	144 (21.6)	0.028
Antiplatelet use	450 (59.3)	59 (64.8)	391 (58.5)	0.251
Single antiplatelet	147 (19.4)	18 (19.8)	129 (19.3)	0.915
Dual antiplatelet	303 (39.9)	41 (45.1)	262 (39.3)	0.286
Anticoagulation use	435 (57.3)	52 (57.1)	383 (57.3)	0.972
Warfarin	242 (31.9)	27 (29.7)	215 (32.2)	0.629
Direct oral anticoagulant	174 (22.9)	22 (24.2)	152 (22.8)	0.762
Heparin	19 (2.5)	3 (3.3)	16 (2.4)	0.489
Combining antiplatelet and anticoagulation therapy	128 (16.9)	20 (22.0)	108 (16.2)	0.165

CAP: complex aortic plaque; IQR: interquartile range; IV: intravenous; mRS: modified Rankin Scale; NIHSS: National Institutes of Health Stroke Scale; tPA: tissue-type plasminogen activator.

### Early ischemic stroke recurrence and functional outcomes at 3 months

**Figure 1:**
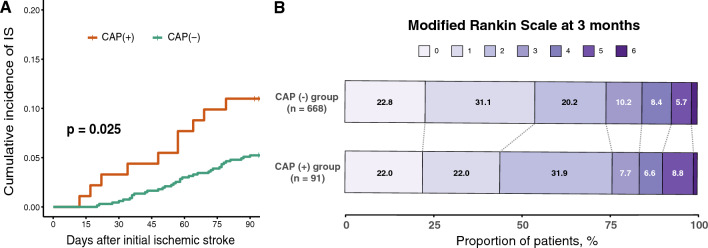
Cumulative incidence of ischemic stroke recurrence stratified by the presence of CAP (**A**) and distribution of modified Rankin Scale at 3 months according to the presence of CAP (**B**) Cumulative curves of ischemic stroke recurrence from index stroke to 90 days stratified by presence of CAP (**A**). Percent distribution of modified Rankin Scale at 3 months (**B**). IS: ischemic stroke; CAP: complex aortic plaque.

Early ischemic stroke recurrence within 3 months was associated with CAP in the short-term cumulative incidence curve (log-rank test, *p* = 0.025, Fig. 1A). However, the proportion of favorable outcomes was not significantly different between patients with CAP (75.9%) and those without (74.1%) (*p* = 0.720, Fig. 1B). In the multivariable binary logistic regression analyses, CAP was not significantly associated with the functional outcome at 3 months (adjusted OR = 1.01, 95% CI = 0.57–1.84, *p* = 0.973, Supplementary Table 2).

### Long-term outcomes of patients with CAP

**Figure 2:**
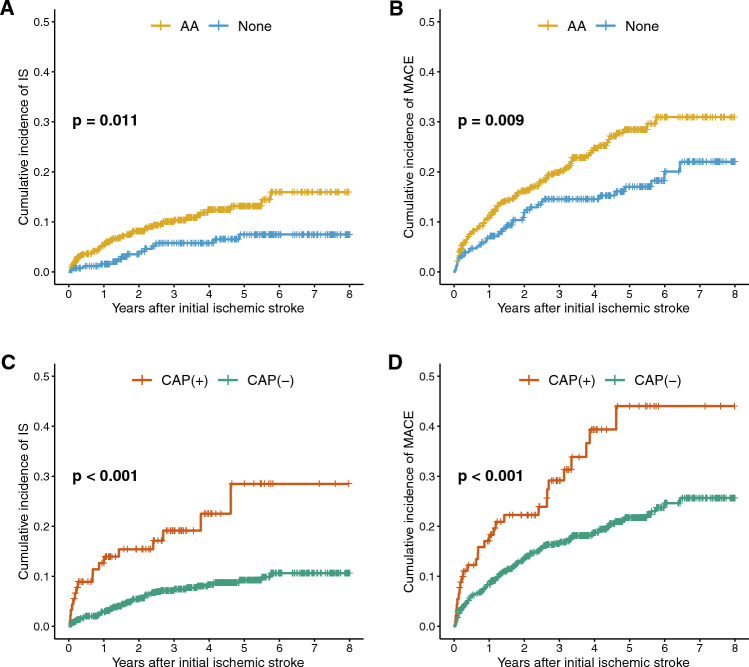
Long-Term Outcomes according to the presence of AA or CAP Cumulative recurrence of ischemic stroke according to the presence of AA (**A**), cumulative occurrence of MACE according to the presence of AA (**B**), cumulative recurrence of ischemic stroke according to the presence of CAP (**C**), and cumulative occurrence of MACE according to the presence of CAP (D). AA: aortic atheroma; CAP: complex aortic plaque; IS: ischemic stroke; MACE: major adverse cardiovascular event.

The median follow-up period was 3.02 (IQR 1.36–4.84) years with a total follow-up of 2,456 person-years. The recurrence rate of ischemic stroke in patients with CAP was 7.4 per 100 person-years, which was significantly higher than the rate of 2.1 per 100 person-years in patients without CAP (*p* < 0.001). Similarly, the MACE occurrence rate was higher in patients with CAP at 12.2 per 100 person-years compared to 5.3 per 100 person-years in those without CAP (*p* < 0.001). The Kaplan–Meier analyses showed that AA was associated with ischemic stroke recurrence (*p* = 0.011) and MACE occurrence (*p* = 0.009, Fig. 2). Among AA, CAP was significantly associated with ischemic stroke recurrence (*p* < 0.001) and MACE occurrence (*p* < 0.001, Fig. 2). In the Cox regression model, the presence of CAP was significantly associated with ischemic stroke recurrence (hazard ratio [HR] = 2.68, 95% CI = 1.48–4.88, *p* = 0.001) and MACE occurrence (HR = 1.61, 95% CI = 1.03–2.51, *p* = 0.039) after adjusting for other significantly associated factors in univariable analysis (Table 3 and 4).

**Table 3:** Univariable and multivariable analyses of ischemic stroke recurrence.

	Univariable analysis HR (95% CI)	*p*-value	Multivariable analysis HR (95% CI)	*p*-value
Age (per 1-year increase)	1.04 (1.01–1.06)	0.001	1.02 (1.00–1.05)	0.048
Sex (women)	1.07 (0.65–1.75)	0.795		
Hypertension	1.33 (0.76–2.31)	0.313		
Diabetes	1.11 (0.65–1.89)	0.710		
Dyslipidemia	1.48 (0.81–2.72)	0.206		
Coronary artery disease	1.11(0.65–1.89)	0.703		
Atrial fibrillation	1.33 (0.81–2.19)	0.252		
Previous ischemic stroke	1.38 (0.72–2.64)	0.328		
Previous hemorrhagic stroke	1.95 (0.71–5.37)	0.196		
Malignancy	1.17 (0.53–2.57)	0.692		
Current smoker	0.53 (0.24–1.15)	0.108		
Pre-stroke mRS (2–5)	4.29 (2.04–9.03)	< 0.001	3.63 (1.70–7.74)	< 0.001
Initial NIHSS score	1.03 (0.99–1.07)	0.154		
IV-tPA	0.83 (0.38–1.83)	0.649		
Endovascular treatment	1.13 (0.56–2.29)	0.730		
Antiplatelet use	1.20 (0.72–1.99)	0.483		
Anticoagulation use	1.19 (0.72–1.95)	0.502		
CAP	3.16 (1.82–5.52)	< 0.001	2.68 (1.48–4.88)	0.001

CAP: complex aortic plaque; CI: confidence interval; HR: hazard ratio; IV: intravenous; mRS: modified Rankin Scale; NIHSS: National Institutes of Health Stroke Scale; tPA: tissue-type plasminogen activator.

**Table 4:** Univariable and multivariable analyses of MACE occurrence.

	Univariable analysis HR (95% CI)	*p*-value	Multivariable analysis HR (95% CI)	*p*-value
Age (per 1-year increase)	1.04 (1.03–1.06)	< 0.001	1.03 (1.02–1.05)	< 0.001
Sex (women)	0.73 (0.52–1.03)	0.078	0.67 (0.47–0.97)	0.034
Hypertension	1.42 (0.98–2.06)	0.064	0.98 (0.66–1.46)	0.931
Diabetes	1.90 (1.37–2.65)	< 0.001	1.58 (1.13–2.23)	0.008
Dyslipidemia	1.37 (0.90–2.08)	0.140		
Coronary artery disease	1.36 (0.97–1.92)	0.074	1.15 (0.81–1.64)	0.429
Atrial fibrillation	1.50 (1.08– 2.09)	0.017	0.95 (0.54–1.67)	0.853
Previous ischemic stroke	1.59 (1.05–2.40)	0.027	1.22 (0.80–1.86)	0.362
Previous hemorrhagic stroke	1.46 (0.68–3.13)	0.326		
Malignancy	2.25 (1.48–3.42)	< 0.001	1.94 (1.27–2.97)	0.002
Current smoker	0.71 (0.44–1.14)	0.155		
Pre-stroke mRS (2–5)	4.18 (2.51–6.94)	< 0.001	3.28 (1.92–5.60)	< 0.001
Initial NIHSS score	1.02 (1.00–1.05)	0.088	1.03 (1.00–1.06)	0.052
IV-tPA	0.60 (0.33–1.08)	0.091	0.55 (0.29–1.04)	0.064
Endovascular treatment	0.92 (0.55–1.52)	0.737		
Antiplatelet use	0.88 (0.64–1.23)	0.461		
Anticoagulation use	1.49 (1.06–2.10)	0.021	1.28 (0.72–2.27)	0.402
CAP	2.10 (1.39–3.17)	< 0.001	1.61 (1.03–2.51)	0.039

CAP: complex aortic plaque; CI: confidence interval; HR: hazard ratio; IV: intravenous; MACE: major adverse cardiovascular events; mRS: modified Rankin Scale; NIHSS: National Institutes of Health Stroke Scale; tPA: tissue-type plasminogen activator.

### Subgroup analyses associated with ischemic stroke recurrence and MACE

There were no significant interactions in any of the subgroups with respect to the outcome of ischemic stroke recurrence and MACE occurrence. The point estimates across all strata indicated an association between CAP and poor long-term outcomes (Supplementary Fig. 2).

### The relationship between use of antithrombotic and long-term outcomes in patients with CAP

**Figure 3:**
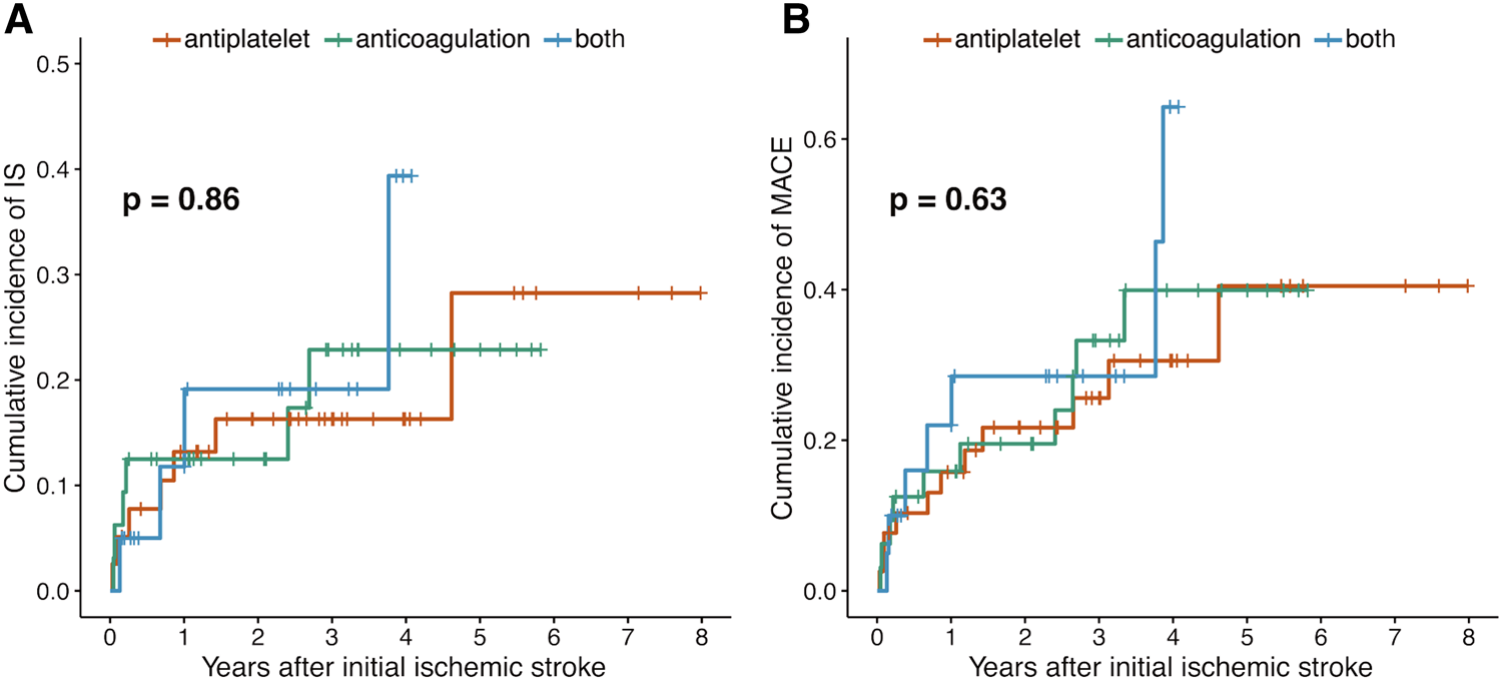
Antithrombotic drugs and long-term outcomes in patients with CAP Cumulative recurrence of ischemic stroke according to the antithrombotic drugs (**A**) and cumulative occurrence of MACE according to the antithrombotic drug (**B**). CAP: complex aortic plaque; IS: ischemic stroke; MACE: major adverse cardiovascular event.

Among patients diagnosed with CAP, 39 (42.9%) were on antiplatelet therapy, 32 (35.1%) were receiving anticoagulation therapy, and 20 (22.0%) were using both types of antithrombotic agents. Patients with antiplatelet were less likely to experience atrial fibrillation and receive endovascular treatment. Patients with both types of antithrombotic agents were more likely to have coronary artery disease and a higher initial NIHSS score (Supplementary Table 3). The cumulative curves representing both ischemic stroke recurrence and MACE occurrence did not indicate any significant difference among groups of antithrombotic agent use (for ischemic stroke recurrence, *p* = 0.86; for MACE occurrence, *p* = 0.63, Fig. 3).

## Discussion

In this study, CAP was associated with poor long-term outcomes including ischemic stroke recurrence and MACE occurrence in patients with cardioembolic stroke. While early ischemic stroke recurrence was higher in patients with CAP than those without CAP, the functional outcome at 3 months was not different.

We found that CAP was independently associated with ischemic stroke recurrence (HR = 2.68) and MACE occurrence (HR = 1.61) in patients with cardioembolic stroke. There were early and late ischemic stroke recurrence after 3 years in Kaplan–Meier curve. Presence of CAP may increase the risk of cerebral embolism in cardioembolic stroke^10^. The mechanism linking CAP to vascular events in cardioembolic stroke patients remains unclear. We hypothesized several mechanisms. First, CAP could be a direct source of embolism. A transcranial Doppler study showed a higher detection rate of microembolic signals in patients with AA^10^. Other studies showed that mobile or ulcerative AA is strongly associated with the recurrence of ischemic stroke^4,11^. These findings indicated that CAP may be an independent source of embolism and increased embolic risk in patients with cardioembolic stroke. Second, AA is associated with concomitant large artery atherosclerosis. One study revealed an association between AA and cervicocephalic artery atherosclerosis, while we also reported a higher prevalence of AA in patients with intracranial artery atherosclerosis^12,13^. Larger atherosclerotic burdens in cardioembolic stroke might be associated with ischemic stroke recurrence other than a cardioembolic mechanism. Ischemic strokes commonly recurred soon after the initial event, with the incidence of late recurrence rising sharply in the CAP group. While we do not have a precise explanation for these findings, we observed a second surge in recurrence after 3 years. The median age of CAP patients was higher compared to non-CAP patients, which suggests that age might influence recurrence rates as it is a significant long-term prognostic factor. Another possible explanation could be the small sample size of CAP patients, as late recurrences were relatively fewer than early ones.

We found that patients with CAP had a higher rate of early ischemic stroke recurrence compared to those without CAP. However, the functional outcomes after 3 months were comparable for the two groups. We postulate reasons for this below. First, patients both with and without CAP had mild strokes (median NIHSS score was 3). In addition, approximately two-thirds of the study population showed good functional outcomes. Mild strokes typically lead to substantial functional recovery. Second, we reported that the MRI lesion pattern resulting from AA exhibited multiple small cortical lesions, which differed from the MRI pattern of cardioembolism^14^. Due to their small volume, multiple small embolic infarctions tend to exhibit mild clinical manifestation. Third, the proportion of patients who were already taking antiplatelet or statin prior to the index stroke was higher in patients with CAP compared to those without CAP. It has been reported that antithrombotic agents and/or statin use prior to the index stroke was independently associated with lower stroke severity^15,16^.

Adequate antithrombotic therapy for patients with CAP remains controversial^17^. The Aortic Arch Related Cerebral Hazard (ARCH) Trial investigated the superiority of aspirin plus clopidogrel over warfarin therapy. During a median follow-up of 3.4 years, recurrent stroke or vascular events were observed in 7.6% of patients on aspirin plus clopidogrel and 11.3% of patients on warfarin, with no significant difference between the treatments^17^. While the current study was not a randomized trial designed to evaluate appropriate antithrombotic therapy, we found no statistical disparity in long-term outcomes according to the type of antithrombotic medication used. These findings align with the results from the previous ARCH trial. Further study is required to determine the optimal use of antithrombotic agents in patients diagnosed with both cardioembolic sources and CAP. A study showed that statins improved the long-term prognosis in patients with cardioembolic stroke^18^. The effects of statins on hidden CAP may contribute to the observed benefits. Statins inhibit cholesterol synthesis and increase LDL receptor synthesis, subsequently reducing serum LDL cholesterol level^19^. These effects result in the modification of lipid metabolism and promote plaque regression, potentially yielding a favorable impact on patients with CAP^20^.

TEE serves as the gold standard for identifying both cardiac embolism and aortic sources of embolism in ischemic stroke^21^. Using TEE, multiple embolic sources are often simultaneously detected in patients with embolic stroke^22^. Patients with cardioembolism (especially atrial fibrillation) are often diagnosed without undergoing TEE. Not conducting a TEE assessment might lead to undiagnosed, hidden CAP. Based on our study, CAP is an independent factor associated with recurrent stroke and vascular events in patients with cardioembolic stroke. TEE could be helpful in evaluating CAP and estimating prognosis.

There are several limitations to this study. First, although we conduct TEE as a standard evaluation, selection bias may exist because not all patients with cardioembolic stroke underwent TEE. Second, TEE is unable to completely assess the aorta due to a blind spot in the upper portion of the ascending aorta, where the air-filled trachea lies between the esophagus and the aorta, hindering visualization^23^. Third, there were no available data of follow-up TEE to identify the effects of changes in CAP morphology or size on ischemic stroke recurrence. Future research that monitors CAP change through follow-up TEE will aid in elucidating the cause of recurrent stroke in patients with CAP. Finally, because this study is a retrospective observational study, the causative relationship between a CAP and the index stroke or recurrence of ischemic stroke could not be determined.

In patients with cardioembolic stroke, CAP was associated with poor long-term outcomes including ischemic stroke recurrence and MACE occurrence. It might be reasonable to consider TEE in the diagnostic workup in patients with cardioembolic stroke to detect CAP.

## Methods

### Study population and evaluation

The Institutional Review Board of Severance Hospital, Yonsei University Health System, approved this study and waived the need for patient-informed consent due to the retrospective design and observational nature (4–2023-0057). Patients for this study were recruited from the Yonsei Stroke Registry^24^. The Registry prospectively enrolled consecutive patients with acute ischemic stroke or transient ischemic attack within 7 days after symptom onset. This study was conducted ethically in accordance with the guidelines of the Declaration of Helsinki.

Stroke etiologies were classified according to the Trial of Org 10,172 in Acute Stroke Treatment (TOAST) classification^25^. These classifications were made at weekly stroke conferences, and any disagreements were resolved by a consensus of three stroke neurologists. The cardioembolic stroke group included patients with acute ischemic stroke who had high- or medium-risk potential cardiac sources of embolism (PCSE) according to the TOAST classification. Patients with significant (> 50%) stenosis or occlusion of the artery relevant to the ischemic stroke, small artery occlusion, and rare cause of stroke (e.g., cerebral artery dissection, cancer stroke, anti-phospholipid syndrome, vasculitis, moyamoya disease) were excluded. In patients with multiple admissions, only data for the first admission during the study period were included.

### TEE and diagnosis of aortic atheroma

TEE was a routine evaluation in the study hospital, and it was performed within two weeks of ischemic stroke onset for all patients, except for those with altered consciousness, impending cerebral herniation, endotracheal intubation, unstable vital signs, or absence of informed consent. A commercially available TEE machine (iE33 xMATRIX, Philips, Andover, MA; or Acuson SC2000, Siemens, Mountain View, CA) equipped with a multiplane 5 MHz transducer was used. Assessment of the aortic arch, ascending aorta, and descending aorta was conducted to detect the presence of AA. The wall thickness exceeding 1 mm was classified as AA^3^. The study utilized the largest diameter AA for classification. CAP was defined as a plaque in the aortic arch or ascending aorta that protruded at least 4 mm or any plaque regardless of size that has a mobile or ulcerative lesion. The TEE results were interpreted and agreed upon by two cardiologists.

### Risk factors and variables

We collected data on demographics and risk factors including age, sex, hypertension, diabetes mellitus, dyslipidemia, coronary artery disease, malignancy, atrial fibrillation, smoking history, continuous cardiac rhythm monitoring, and past stroke history. We also collected (i) the National Institutes of Health Stroke Scale (NIHSS) score at admission, (ii) the pre-stroke modified Rankin Scale (mRS) score to confirm the severity of the initial ischemic stroke and functioning prior to the stroke, (iii) the use of intravenous tissue type plasminogen activator, (iv) the presence of endovascular treatment, and (v) previous history of antithrombotic or statin use. We additionally investigated the use of antithrombotic and statin before and after the index stroke. Antithrombotic use after the index stroke was categorized into three groups: those using antiplatelet, those on anticoagulation, and those taking both types of agents.

### Outcomes

Patients were managed according to current guidelines during the study period^26,27^. The short-term functional outcome was determined by the mRS score at 3 months. We divided patients into two groups based on their mRS score at 3 months: those with favorable outcomes (mRS score of 0–2) and those with unfavorable outcomes (mRS score of 3–6). To identify the association between early ischemic stroke recurrence and CAP, the short-term cumulative incidence of ischemic stroke was investigated for up to 3 months. Long-term outcomes were assessed by the recurrence of ischemic stroke and the occurrence of MACE. Recurrence of ischemic stroke was defined as a new ischemic stroke occurring at least 7 days after the index event. MACE was defined as any events of ischemic stroke recurrence, hemorrhagic stroke occurrence, acute coronary syndromes, or all causes of death. After being discharged from the hospital, all patients were periodically followed and scheduled to visit the outpatient clinic. At each follow-up visit, we investigated the mRS score, the recurrence of ischemic stroke, and the occurrence of MACE. When patients missed the scheduled visit, information was obtained from telephone interviews by trained stroke nurses with structured questionnaires. The censoring date was December 31, 2019.

### Statistical analyses

Continuous variables were reported as median (interquartile range [IQR]) and categorical variables as number (%). The Kruskal–Wallis test, Wilcoxon rank sum test, chi-square test, and Fisher’s exact test were appropriately used for comparing baseline characteristics. Variance inflation factor was used to confirm multicollinearity among confounders. For functional outcomes, univariable binary logistic regression analyses were performed to calculate odd ratios (ORs) and corresponding 95% confidence intervals (CIs).

Multivariable binary logistic regression analyses were performed to adjust for statistically significant variables in univariable analyses (*p*-value < 0.1) and the presence of CAP. For long-term outcomes, Kaplan–Meier analyses with log-rank tests were used to investigate the cumulative incidence of ischemic stroke recurrence and MACE occurrence. Multivariable Cox proportional hazard regression analyses were performed using the variables with a *p*-value < 0.1 in the univariable analysis.

Subgroup analyses of the long-term outcomes were analyzed using the Cox proportional hazard regression model according to age (> 70 and ≤ 70 years, based on the median value of study population), sex, hypertension, diabetes mellitus, atrial fibrillation, initial NIHSS score (> 4 and ≤ 4, based on the median value of study population), treatment with intravenous tissue plasminogen activator, endovascular treatment, and high or medium risk PCSE. The *p*-value for interactions was calculated to investigate the consistency of the main finding among the subgroups.

A two-sided *p*–value < 0.05 was considered statistically significant. All data were analyzed using R version 4.2.2 (R Foundation for Statistical Computing, Vienna, Austria). This study followed the Strengthening the Reporting of Observational studies in Epidemiology (STROBE) guidelines^28^.

### Supplementary Information

Supplementary Information.

## Data Availability

The supporting data are available from the corresponding author on reasonable request.
