# Supplementary material for: Prognosis of ischemic stroke patients with both aortic atheroma and cardioembolic sources
Source: Sci Rep. 2024 Jun 3;14:12656. doi: 10.1038/s41598-024-60294-1 (PMC11144702; doi:10.1038/s41598-024-60294-1)
Supplement: Supplementary file 1 — Supplementary Information. [file 41598_2024_60294_MOESM1_ESM.docx]

# Supplementary Materials

# Prognosis of ischemic stroke patients with both aortic atheroma and cardioembolic sources

**Jae Wook Jung, Hyo Suk Nam, et al.**

## Supplementary Fig. 1. Flow diagram of study population according to inclusion and exclusion criteria

Supplementary Fig. 2**.** Subgroup analyses of adjusted hazard ratios of CAP for long-term outcomes

Supplementary Table 1. Comparison of patients with TEE to those without

## Supplementary Table 2. Univariable and multivariable binary logistic regression analysis for good functional outcome (mRS 0–2) at 3 months

# Supplementary Table 3. Baseline characteristics of study population with CAP according to antithrombotic drugs

## **Supplementary Fig 1. Flow diagram of study population according to inclusion and exclusion criteria**

Abbreviations: TEE = transesophageal echocardiography.

**Supplementary Fig 2. Subgroup analyses of adjusted hazard ratios of CAP for long-term outcomes**

**
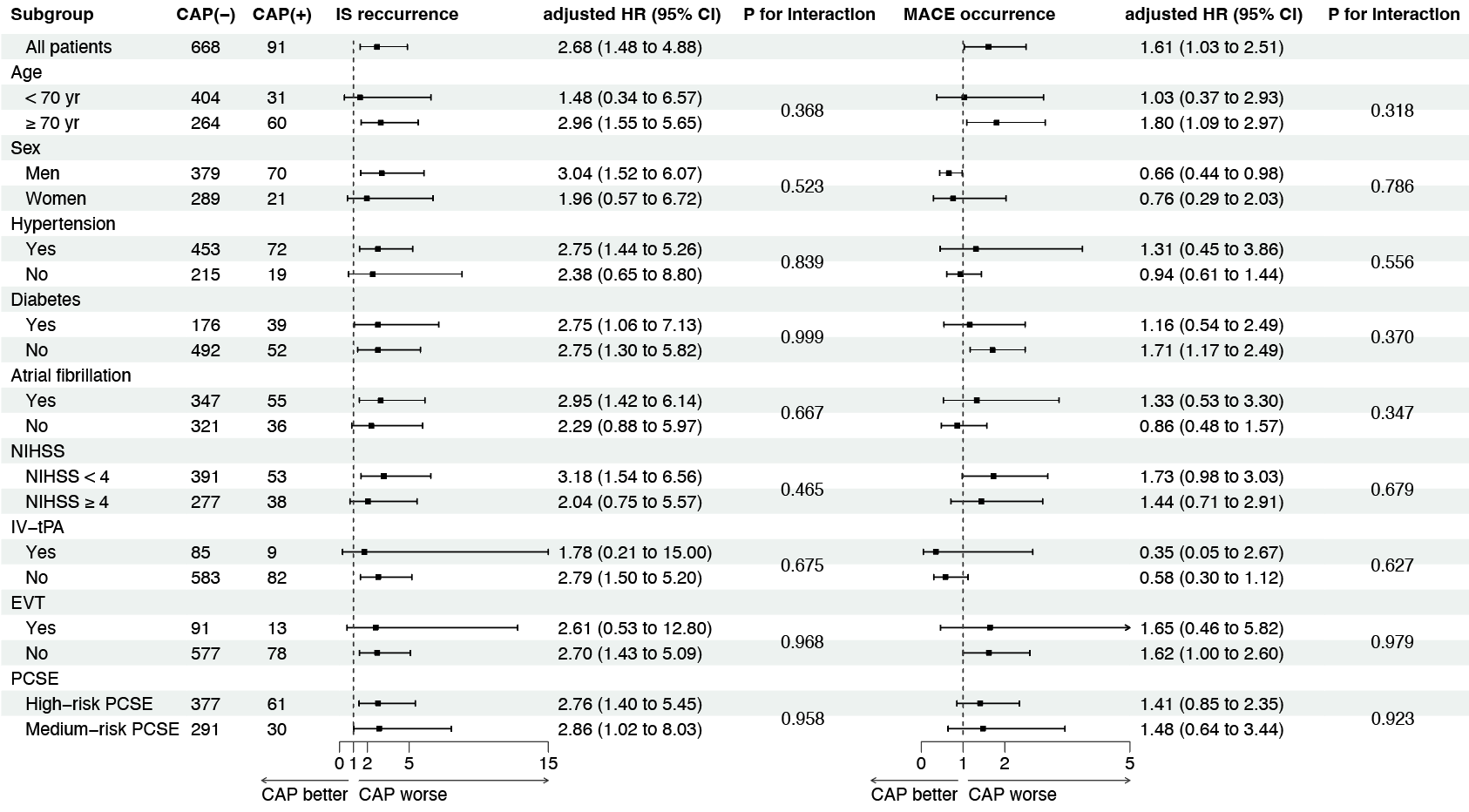
**

Subgroup analyses of IS recurrence adjusted for age, pre-stroke mRS (2-5), initial NIHSS score, and CAP. Subgroup analyses of MACE occurrence adjusted for age, sex, hypertension, diabetes, coronary artery disease, atrial fibrillation, malignancy, pre-stroke mRS (2-5), initial NIHSS score, IV-tPA, anticoagulation use, and CAP.

Abbreviations: CAP = complex aortic plaque; EVT = endovascular treatment; HR = hazard ratio; IS = ischemic stroke; IV = intravenous; MACE = major adverse cardiovascular events; mRS = modified Rankin Scale; NIHSS = National Institutes of Health Stroke Scale; PCSE = potential cardiac source of embolism; tPA = tissue-type plasminogen activator.

**Supplementary Table 1. Comparison of patients with TEE to those without**

|  | Total  (n = 1,207) | TEE (+)  (n = 759) | TEE (-)  (n = 448) | *p*-value |
| --- | --- | --- | --- | --- |
| **Demographic variables** |  |  |  |  |
| Age (years), median (IQR) | 73 (61, 81) | 68 (57, 75) | 81 (74, 86) | <0.001 |
| Sex (women), n (%) | 522 (43.2) | 310 (40.8) | 212 (47.3) | 0.028 |
| Hypertension | 868 (72.0) | 525 (69.2) | 343 (76.7) | 0.005 |
| Diabetes | 373 (30.9) | 215 (28.3) | 158 (35.3) | 0.011 |
| Dyslipidemia | 205 (17.0) | 119 (15.7) | 86 (19.3) | 0.110 |
| Coronary artery disease | 312 (25.9) | 209 (27.5) | 103 (23.0) | 0.085 |
| Malignancy | 148 (12.3) | 83 (10.9) | 65 (14.6) | 0.061 |
| Atrial fibrillation | 762 (63.2) | 402 (53.0) | 360 (80.5) | <0.001 |
| Current smoker | 182 (15.1) | 136 (17.9) | 46 (10.3) | <0.001 |
| Cardiac rhythm monitoring | 975 (90.5) | 639 (90.5) | 336 (90.6) | 0.999 |
| Previous ischemic stroke | 204 (16.9) | 104 (13.7) | 100 (22.3) | <0.001 |
| Previous hemorrhagic stroke | 56 (4.6) | 27 (3.6) | 29 (6.5) | 0.020 |
| **Stroke characteristics** |  |  |  |  |
| Initial NIHSS score | 4 (1, 12) | 3 (1, 7) | 10 (2, 18) | <0.001 |
| IV-tPA | 156 (12.9) | 94 (12.4) | 62 (13.8) | 0.495 |
| Endovascular treatment | 216 (17.9) | 104 (13.7) | 112 (25.0) | <0.001 |
| Pre-stroke mRS (2–5) | 93 (8.1) | 33 (4.3) | 60 (15.6) | <0.001 |
| Antiplatelet, prior to admission | 412 (34.2) | 246 (32.4) | 166 (37.1) | 0.095 |
| Anticoagulation, prior to admission | 235 (19.5) | 128 (16.9) | 107 (23.9) | 0.003 |
| Statin, prior to admission | 312 (25.9) | 173 (22.8) | 139 (31.1) | 0.001 |
| Antiplatelet use | 641 (53.2) | 450 (59.3) | 191 (42.7) | <0.001 |
| Anticoagulation use | 730 (60.5) | 435 (57.3) | 295 (66.0) | 0.003 |

Values are presented as number (%) or median (IQR).

Abbreviations: IQR = interquartile range; IV = intravenous; mRS = modified Rankin Scale; NIHSS = National Institutes of Health Stroke Scale; TEE = transesophageal echocardiography; tPA = tissue-type plasminogen activator.

**Supplementary Table 2. Univariable and multivariable binary logistic regression analysis for good functional outcome (mRS 0–2) at 3 months**

|  | Univariable analysis  OR (95% CI) | *p*-value | Multivariable analysis  OR (95% CI) | *p*-value |
| --- | --- | --- | --- | --- |
| Age (per 1-year increase) | 0.98 (0.97–1.00) | 0.010 | 0.99 (0.98–1.01) | 0.209 |
| Sex (women) | 0.69 (0.49–0.95) | 0.024 | 0.87 (0.59–1.28) | 0.495 |
| Hypertension | 0.79 (0.55–1.13) | 0.201 |  |  |
| Diabetes | 0.88 (0.62–1.27) | 0.488 |  |  |
| Dyslipidemia | 0.82 (0.53–1.28) | 0.312 |  |  |
| Coronary artery disease | 1.06 (0.74–1.54) | 0.753 |  |  |
| Atrial fibrillation | 0.81 (0.58–1.12) | 0.199 |  |  |
| Previous ischemic stroke | 0.88 (0.56–1.41) | 0.582 |  |  |
| Previous hemorrhagic stroke | 0.49 (0.22–1.10) | 0.074 | 0.51 (0.22–1.23) | 0.125 |
| Malignancy | 0.64 (0.40–1.05) | 0.072 | 0.60 (0.35–1.06) | 0.072 |
| Current smoker | 1.21 (0.79–1.90) | 0.394 |  |  |
| Initial NIHSS score | 0.85 (0.83–0.88) | <0.001 | 0.81 (0.77–0.84) | <0.001 |
| IV-tPA | 1.23 (0.75–2.11) | 0.428 |  |  |
| Endovascular treatment | 0.55 (0.36–0.85) | 0.007 | 4.53 (2.38–8.95) | <0.001 |
| Antiplatelet use | 0.88 (0.63–1.23) | 0.458 |  |  |
| Anticoagulation use | 0.86 (0.62–1.20) | 0.379 |  |  |
| CAP | 1.10 (0.67–1.86) | 0.724 | 1.01 (0.57–1.84) | 0.973 |

Abbreviations: CAP = complex aortic plaque; CI = confidence interval; OR = odds ratio; IV = intravenous; mRS = modified Rankin Scale; NIHSS = National Institutes of Health Stroke Scale; tPA = tissue-type plasminogen activator.

# Supplementary Table 3. Baseline characteristics of study population with CAP according to antithrombotic drugs

|  | Antiplatelet  (n = 39) | Anticoagulation (n = 32) | Both  (n = 20) | *p-*value |
| --- | --- | --- | --- | --- |
| **Demographic variables** |  |  |  |  |
| Age (years), median (IQR) | 76 (69, 81) | 75 (64, 79) | 74 (68, 79) | 0.641 |
| Sex (women), n (%) | 7 (17.9) | 10 (31.3) | 4 (20.0) | 0.428 |
| Hypertension | 31 (79.5) | 25 (78.1) | 16 (80.0) | 0.999 |
| Diabetes | 18 (46.2) | 12 (37.5) | 9 (45.0) | 0.746 |
| Dyslipidemia | 6 (15.4) | 10 (31.3) | 10 (10.0) | 0.125 |
| Coronary artery disease | 11 (28.2) | 9 (28.1) | 12 (60.0) | 0.031 |
| Malignancy | 4 (10.3) | 3 (9.4) | 1 (5.0) | 0.899 |
| Atrial fibrillation | 7 (17.9) | 32 (100) | 16 (80.0) | <0.001 |
| Current smoker | 11 (28.2) | 5 (15.6) | 3 (15.0) | 0.360 |
| Cardiac rhythm monitoring | 33 (86.8) | 25 (86.2) | 17 (100) | 0.375 |
| Previous ischemic stroke | 8 (20.5) | 4 (12.5) | 4 (20.0) | 0.724 |
| Previous hemorrhagic stroke | 1 (2.6) | 1 (3.1) | 2 (10.0) | 0.431 |
| **Stroke characteristics** |  |  |  |  |
| Pre-stroke mRS (2–5) | 0 (0.0) | 1 (3.1) | 2 (10.0) | 0.100 |
| Initial NIHSS score, median (IQR) | 2 (1, 4) | 2.5 (1, 7) | 7 (4, 12) | 0.001 |
| IV-tPA | 3 (7.7) | 4 (12.5) | 2 (10.0) | 0.901 |
| Endovascular treatment | 1 (2.6) | 8 (25.0) | 4 (20.0) | 0.009 |
| Antiplatelet, prior to admission | 17 (43.6) | 13 (40.6) | 9 (45.0) | 0.946 |
| Anticoagulation, prior to admission | 1 (2.6) | 10 (31.3) | 4 (20.0) | 0.002 |
| Statin, prior to admission | 11 (28.2) | 13 (40.6) | 5 (25.0) | 0.405 |

Values are presented as number (%) or median (IQR).

Abbreviations: CAP = complex aortic plaque; HDL = high-density lipoprotein; IQR = interquartile range; IV = intravenous; LDL = low-density lipoprotein; mRS = modified Rankin Scale; NIHSS = National Institutes of Health Stroke Scale; tPA = tissue-type plasminogen activator.
